# Synthesis and Characterization of a Multi-Walled Carbon Nanotube–Ionic Liquid/Polyaniline Adsorbent for a Solvent-Free In-Needle Microextraction Method

**DOI:** 10.3390/molecules28083517

**Published:** 2023-04-17

**Authors:** Soyoung Ahn, Sunyoung Bae

**Affiliations:** Department of Chemistry, Seoul Women’s University, 621 Hwarang-ro, Nowon-gu, Seoul 01797, Republic of Korea; ahnso7179@naver.com

**Keywords:** polyaniline, in-needle microextraction, multi-walled carbon nanotube, ionic liquid, electrochemical polymerization

## Abstract

Sample preparation is an essential process when handling complex matrices. Extraction without using a solvent requires the direct transfer of analytes from the sample to the adsorbent either in the gas or liquid phase. In this study, a wire coated with a new adsorbent was fabricated for in-needle microextraction (INME) as a solvent-free sample extraction method. The wire inserted into the needle was placed in the headspace (HS), which was saturated with volatile organic compounds from the sample in a vial. A new adsorbent was synthesized via electrochemical polymerization by mixing aniline with multi-walled carbon nanotubes (MWCNTs) in the presence of an ionic liquid (IL). The newly synthesized adsorbent using IL is expected to achieve high thermal stability, good solvation properties, and high extraction efficiency. The characteristics of the electrochemically synthesized surfaces coated with MWCNT–IL/polyaniline (PANI) adsorbents were characterized using Fourier transform infrared (FTIR) spectroscopy, scanning electron microscopy (SEM), thermogravimetric analysis (TGA), and atomic force microscopy (AFM). Then, the proposed HS–INME–MWCNT–IL/PANI method was optimized and validated. Accuracy and precision were evaluated by analyzing replicates of a real sample containing phthalates, showing spike recovery between 61.13% and 108.21% and relative standard deviations lower than 15%. The limit of detection and limit of quantification of the proposed method were computed using the IUPAC definition as 15.84~50.56 μg and 52.79~168.5 μg, respectively. We concluded that HS–INME using a wire coated with the MWCNT–IL/PANI adsorbent could be repeatedly used up to 150 times without degrading its extraction performance in an aqueous solution; it constitutes an eco-friendly and cost-effective extraction method.

## 1. Introduction

Chemical analysis involves several steps, including sampling, extraction purification, and sample introduction [1,2,3]. Major issues are caused by interferences and low concentrations of analytes in the sample matrix. An extraction procedure may be required to separate and enrich the target analyte from the complicated matrixes [4]. The extraction methods used for sample analysis in the aqueous phase are liquid–liquid extraction, solid-phase extraction, solid phase microextraction, and in-needle microextraction [5,6,7,8,9,10,11].

In-needle microextraction (INME) was developed as a simple sample preparation method [11,12,13,14,15,16]. To fabricate the INME needle, a layer of adsorbent is coated inside the needle or outside the wire to be inserted into the needle [11,12,13,14,15,16]. The fabricated needle is attached to a syringe and directly placed in an aqueous solution or exposed in the headspace of the vial containing the analytes during the extraction process. The adsorbent can be synthesized using sol-gel polymerization [12,13,14] or electrochemical deposition [11,15,16,17].

Among the various adsorbents containing activated carbon, neutral zeolites, alumina, biochar, and clay minerals, polymeric substances have attracted a great deal of attention due to their simple synthesis reaction, adjustable morphology, and different functional groups [18]. For INME adsorbents, a polyaniline-based adsorbent has been investigated because it shows conducting properties, good mechanical stability, and high efficiency of adsorption, and it is abundant in nature. The chemical oxidative polymerization of aniline is initiated by adding an oxidant in a strong acidic solution. The mixture of multi-wall carbon nanotubes (MWCNTs) with PANI during synthesis exhibits a large surface area, high electrical conductivity, rich stacking π electrons, high mechanical strength, and outstanding chemical and thermal stability [11,19]. Due to the unique properties of these mixtures, MWCNTs show promise in a variety of applications such as sensors [20], sampling [21], solid-phase extraction [22], solid-phase microextraction (SPME) [23], chromatography [24], and the INME method [11]. However, widespread use of CNTs has been limited due to their agglomeration and weak coating stability [25].

In this study, a new adsorbent, synthesized with MWCNT–IL/PANI, was coated on a stainless-steel wire and inserted into the INME needle. IL was used as an additive to achieve a well-dispersed MWCNT due to its distinct characteristics, which include low toxicity, low melting points, high thermal stability, and good solvation properties [26,27,28]. CNT–IL hybrids are ideal for sampling applications [29,30,31]. The physicochemical properties of the synthesized adsorbent were characterized using Fourier transform infrared (FTIR) spectroscopy, thermogravimetric analysis (TGA), and atomic force microscopy (AFM). For the feasibility study, the HS–INME method followed by GC/MS was applied to hot water covered by low-density polyethylene (LLDPE) wrap, as an example of the real sample. The proposed HS–INME–MWCNT–IL/PANI method was optimized and validated according to metrics including the limit of detection (LOD), limit of quantification (LOQ), dynamic range, recovery, and reproducibility.

## 2. Results and Discussion

### 2.1. Optimization of the HS–INME–MWCNT–IL/PANI Coating Layer

Extraction efficiency can be affected by various parameters, including MWCNT–IL/PANI synthesis and the extraction conditions when using HS-INME followed by GC/MS. Synthesis conditions, such as the mixing ratio of MWCNTs and IL, the polymerization potential, the electrochemical deposition time, and the coating layer’s length, were varied to compare the peak areas as well as standard deviations of the standard compounds. The HS–INME–GC/MS analysis conditions were investigated by changing the saturation time, extraction temperature, and adsorption time.

It can be inferred that the higher the peak area obtained, the higher the adsorption efficiency of the analyte on the adsorbent. The working solution used in the optimization experiment was run in an aqueous solution containing 20 µg mL^−1^ phthalates and all measurements were repeated three times.

#### 2.1.1. Effect of Synthesis Conditions on Extraction

The peak area of phthalates for the HS–INME–MWCNT–IL/PANI method was determined according to the percentage (% *w*/*v*) of MWCNTs and IL within the range of 5% to 15%. As shown in Figure S1, the adsorption of phthalates was largely similar at all rates investigated in this study. However, the standard deviation of the peak areas of 5% and 15% was relatively higher than that of 10%. In addition, impurities were observed at 15%. As a result, 10% MWCNT–IL (% *w*/*v*) was determined to be the optimal mixing ratio.

The polymerization potential was investigated within the range of 1.0 V and 3.0 V. As shown in Figure S2, all phthalates showed higher peak areas at 2.0 V than at 1.0 V and 3.0 V. Even though good reproducibility was shown at 3.0 V, this was not considered to be an optimal condition because the stainless-steel wire might itself react [11]. Therefore, the potential of 2.0 V was determined to be the optimal polymerization potential for coulometry.

The effect of the electrochemical deposition time was investigated from 150 s to 700 s in consideration of the time as well as the extraction efficiency. As shown in Figure S3, the extraction efficiency of the phthalates increased up to 350 s and then decreased at 500 s. The high standard deviation of the peak areas at 150 s and 350 s suggest that this may not be sufficient to solidify the hybrid composite on a wire. Therefore, the optimum deposition time was set to 500 s, which showed the best reproducibility based on the peak area value among the subsequent deposition times.

The effect of adsorbent length on the phthalate extraction efficiency was investigated in the range of 0.5 cm to 1.5 cm. As shown in Figure S4, the longer the coating length, the more phthalates were adsorbed. However, it was noted that the standard deviation of the peak area value was much larger, with a length of more than 1 cm. Therefore, the coating length of 1 cm was set as the optimum condition; similar values have been reported in other studies [11,15,16].

#### 2.1.2. Effect of the Extraction Conditions of the HS–INME Method on Extraction

The extraction efficiency of the phthalates was determined according to the saturation time from 15 min to 60 min. As shown in Figure S5, the adsorption efficiency appeared to be reasonable at the initial saturation time of 15 min, but the standard deviations were very large. After 15 min, the peak areas of all phthalates were shown to be similar, but with different standard deviations. The highest reproducibility was obtained at 60 min saturation, so this was determined to be the optimal saturation time.

For the extraction efficiency of phthalates, the extraction temperature was investigated at various temperatures from 25 °C to 80 °C. Figure S6 shows that the peak area of the phthalates, except for benzyl butyl phthalate and di(2-ethylhexyl) phthalate, which have high molecular weights, is large at 25 °C, with very high standard deviation values. Therefore, the peak area for compounds with a higher molecular weight at 50 °C with a small standard deviation was determined to be the optimal extraction temperature. It is believed that increasing the extraction temperature increases the diffusion coefficient and decreases the distribution constant, resulting in faster equilibrium times than those achieved at room temperature [32].

The adsorption time is a critical parameter for reaching equilibrium in the distribution of analytes between the MWCNT–IL/PANI coating layers and samples in HS. The adsorption time was investigated in the range of 10 min to 60 min. Figure S7 shows that the peak area for all phthalate compounds increases as the adsorption time increases. Even though the adsorption amount was the highest at 60 min, this was not determined to be the optimal condition due to the high standard deviation. As a result, the optimal adsorption time was selected as 30 min, with high peak areas for phthalates.

The effect of the desorption time on the extraction efficiency of the phthalates was evaluated. The desorption percentages for the desorption times of 30 s, 1 min, 3 min, and 5 min at 230 °C were compared, as shown in Figure S8. The phthalate compounds were desorbed by almost 100% as the desorption time increased, except for dibutyl phthalate and di(2-ethylhexyl) phthalate. Finally, the optimized desorption time was set at 3 min.

### 2.2. Synthesis and Characteristics of the MWCNT–IL/PANI Layer on a Wire

Synthesis of the MWCNT–IL/PANI via a number of steps was confirmed by FT–IR and TGA, and coating of the hybrid composite was confirmed by SEM and EDS mapping.

**Figure 1:**
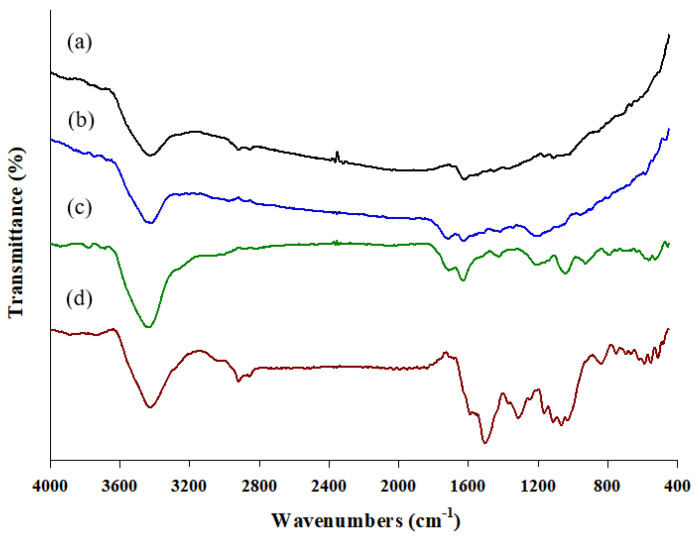
The FT-IR spectra of (a) pristine MWCNTs, (b) oxidized MWCNT, (c) MWCNT–IL, and (d) the MWCNT–IL/PANI composite.

The FT–IR analysis was conducted to confirm that the target functional groups were those generated in the process of combining the MWCNT hybrid composite described in Section 3.2. Figure 1 shows the FT-IR spectra of (a) pristine MWCNTs, (b) oxidized MWCNTs, (c) MWCNT–IL, and (d) the MWCNT–IL/PANI composite.

From the oxidation of the MWCNT composite, a C=C stretching band appeared at 1627.20 cm^−1^; C=O and C–O stretching bands were observed at 1714.69 cm^−1^ and 1215.97 cm^−1^, respectively, due to the carboxyl group, while the pristine MWCNTs showed a C=C stretching peak at 1624.46 cm^−1^. Finally, the MWCNT composite combined with the IL confirmed the presence of C=O and C=C stretching band peaks at 1713.90 cm^−1^ and 1630.56 cm^−1^, respectively; moreover, C–N stretching band peaks from the imidazolium cation were identified through the 1425.58 cm^−1^, 1213.70 cm^−1^, 1045.32 cm^−1^, and 927.63 cm^−1^ vibration bands. As a result, it was confirmed that the MWCNT–IL hybrid composite was successfully synthesized and identified by several major peaks generated in each preparation step.

For the MWCNT–IL/PANI composite at optimum conditions, the benzenoid ring stretching peaks of 1504.68 cm^−1^ and the quinonoid ring stretching peaks from 1591 cm^−1^ were observed in the polyaniline structure, which was oxidized by electrochemical polymerization [33,34]. The in-plane and out-of-plane deformation of C–H bands at 1066.77 cm^−1^, 836.64 cm^−1^, and 752.32 cm^−1^ confirmed the presence of a quinonoid ring [35]. Additionally, we identified a C–N stretching band at 1314.91 cm^–1^ from the secondary aromatic amine [36] and a N–H stretching peak at 590.16 cm^−1^ from the primary amino group [37,38]. The peaks identified in the MWCNT–IL composite and the representative peaks of the PANI were simultaneously identified, indicating that the electrochemically synthesized MWCNT–IL and PANI were successfully cured under optimum conditions.

The TGA was used to determine the thermal stability of MWCNT–IL/PANI, a wire-coated adsorbent at optimum conditions. From Figure S9, it was found that the MWCNT–IL/PANI adsorbent is thermally stable up to 368 °C. Considering that the decomposition temperature of PANI is usually about 200 °C [28,30], thermal stability is improved with the interaction of PANI with MWCNT–IL. This enhancement might be attributed to the long conjugate π–π bond between MWCNT and PANI, similar to that reported in previous studies [11,39,40].

The morphological image of the surface was obtained using AFM (Figure S10). The R_q_ value of the surface was 0.084 nm, while the R_a_ value was 0.038 nm. The root mean square (RMS) roughness (R_q_) refers to the square root of the surface height distribution, which is considered more sensitive than the average roughness (R_a_) for large deviations from the mean line or plane [41]. It can be inferred that the coating of the adsorbent consisting of MWCNT–IL/PANI was evenly spread on the surface of the stainless-steel wire.

**Figure 2:**
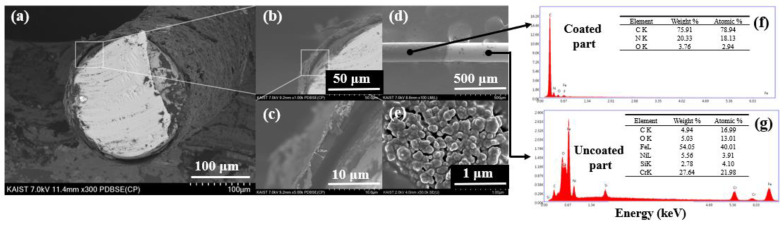
Cross-section SEM images of MWCNT–IL/PANI–coated wire (**a**–**e**) and EDS spectrum of the coated wire (**f**) and uncoated wire with MWCNT–IL/PANI (**g**).

The cross-section image of the MWCNT–IL/PANI coated on a wire in Figure 2 shows that the adsorbent was evenly distributed on the wire with a thickness of about 2.25 μm. In the elemental composition of the uncoated part (Figure 2f), large quantities of Fe (54.05%) and Cr (27.64%) were observed from the stainless-steel part, while the coated part (Figure 2g) consisted of C (75.9%), N (20.33%), and O (3.76%) from MWCNT–IL/PANI. Elemental mapping analysis at the coating interface shows that C, N, and O are evenly distributed on the INME wire.

### 2.3. Validation of the Analytical Method

The adsorption efficiency was investigated using phthalate compounds. The HS–INME–MWCNT–IL/PANI method used in this study was verified by the regression equation of each calibration curve, as well as the LOD, LOQ, and dynamic ranges, and precision and accuracy tests (Table 1). Using the IUPAC definition, the LOD and LOQ were obtained from the mean of the blank measurement and the standard deviation of the blank measurement with a ratio. The values of the ratio were 3 for LOD and 10 for LOQ. Most of phthalates investigated in this study showed very good linearity, with r^2^ = 0.99. Each regression equation of 5 points was calculated by taking measurements three times at each concentration under the optimum conditions. LOD and LOQ were calculated according to the IUPAC definition [42,43], resulting in values of 15.84~50.56 μg for LOD and 52.79~168.5 μg for LOQ, while the dynamic range was between 52.79 μg and 1.00 × 10^3^ μg.

The accuracy of the method was assessed through spike recovery. The sample was investigated by adding 200 μg of phthalate standards. The accuracy results ranged from 61.13% to 108.21%. The identified absolute values and the recovery values obtained by the spikes of the standards are listed in Table S3.

The precision in reproducibility was calculated using the relative standard deviation value. The intra assay (run-to-run) was repeated five times using the same needle, while the inter assay (needle-to-needle) was repeated five times using five different needles. Relative standard deviations for intra assays between the same needles showed an average of less than 10%, and inter-assay experiments comparing five different needles showed an average of less than 15% (Table S3).

The LOD and LOQ of HS–INME–MWCNT–IL/PANI were found to be higher than in the previous study [11,15]. However, the addition of IL might contribute to the homogenous dispersion of MWCNTs in mixture, which could lead to improved recovery and reproducibility compared to HS–INME–MWCNT–PANI [11].

### 2.4. Comparison of Extraction Efficiency

Extraction efficiency was compared according to the dynamic and static INME methods. The difference between static and dynamic HS lies in the presence of a pumping application that can circulate the upper part of the sample during the adsorption process. Phthalates of 200 μg were analyzed under optimized conditions for each method, and the enrichment factor (EF) was calculated using the peak areas of all phthalates investigated in this study (Equation (1)). EF refers to the enriched concentration of the target compound in the adsorbent during the extraction process. Higher EF values indicate better extraction efficiencies than lower EF values.

EF = A_1_/A_0_ (1)

In the equation, A_1_ is the area of the GC/MS peak obtained using the dynamic HS method, and A_0_ is the area of the GC/MS peak obtained using the static HS method. EF values were obtained as 1.60 (±0.11) for dimethyl phthalate, 1.63 (±0.13) for diethyl phthalate, 3.10 (±0.41) for diallyl phthalate, 3.76 (±0.40) for dibutyl phthalate, 4.05 (±0.28) for benzyl butyl phthalate, and 2.61 (±0.33) for di(2-ethylhexyl) phthalate. For all phthalate components, the dynamic HS method has an average EF value of at least 1.5 times that of the static HS method. In particular, benzyl butyl phthalate had the highest value of EF, implying that dynamic extraction with the INME adsorbent synthesized in this study was effective for heavy molecules with benzene substitutes. A higher molecular weight tends to produce higher EF values. However, it was confirmed that the EF value of di(2-ethylhexyl) phthalate does not increase significantly, even though its molecular weight is higher than that of benzyl butyl phthalate. This might contribute to the fact that benzyl butyl phthalate has more benzene ring structures than di(2-ethylhexyl) phthalate, which has a significant effect on producing a better π–π interaction with the MWCNT–IL/PANI used as adsorbent. If the molecular weight is very high, it can be inferred that it may affect the difference between the dynamic and static HS methods. The value of EFs was found to be between 1.60 (for dimethyl phthalate) and 4.05 (for benzyl butyl phthalate).

### 2.5. Application of HS–INME–MWCNT–IL/PANI to an Aqueous Sample

**Figure 3:**
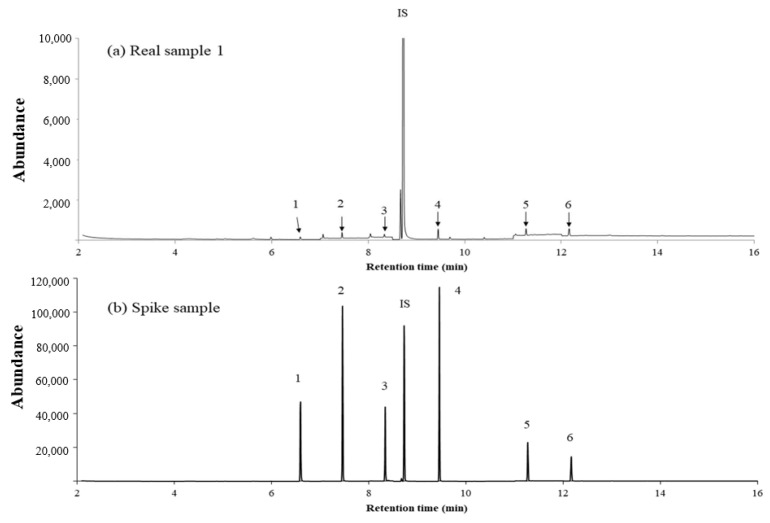
Chromatogram obtained from (**a**) sample 1, and (**b**) spiked sample 1 using HS–INME–MWCNT–IL/PANI. Peak 1, dimethyl phthalate; 2, diethyl phthalate; 3, diallyl phthalate; 4, dibutyl phthalate; 5, benzyl butyl phthalate; 6, di(2-ethylhexyl) phthalate; and IS, anthracene.

The HS–INME–MWCNT–IL/PANI was applied to phthalates in aqueous samples for the feasibility study. To simulate hot water covered by plastic wrap, it was immersed into the water at 80 °C. Phthalates might be leached out into hot water. Figure 3a shows a chromatogram of the phthalates eluted from an industrial LLDPE wrap (sample 1). A similar result for household LLDPE wrap (sample 2) is shown in Figure S11. The measurements of the phthalates are summarized in Table S3. Phthalates from the industrial wrap (sample 1) and a household LLDPE wrap were detected but not quantified due to having lower values than the LOD, except for that of dibutyl phthalate. The values of the dibutyl phthalate concentration were measured as 26.05 μg ± 3.12 μg for the industrial wrap and 24.87 μg ± 1.65 μg for the household LLDPE wrap. Similar data were obtained regarding the migration of plasticizers from LDPE films as food packaging [44]. The chromatogram of the spike sample (Figure 3b) indicated the increase in the peak area by the known amount added. The results showed reasonable recovery and reproducibility, implying that the proposed method could be used as an HS–INME for aqueous samples.

## 3. Materials and Methods

### 3.1. Reagents and Materials

Aniline (>99.5%) as a monomer and nitric acid (64.0~66.0%) were purchased from Duksan Pure Chemical Co. (Ansan, Republic of Korea). The MWNCTs were about 1.0 μm~2.0 μm in length with 10.0 nm in diameter, and the specific surface area was 350 ± 5 m^2^ g^–1^; they were obtained from NTP (Shenzhen, China). A supply of 1-(2-Hydroxyethyl)-3-methylimidazolium tetrafluoroborate ([HOEMIm]BF4) (>98.0%) was purchased from TCI (Tokyo, Japan) and N, N-dimethylformamide (DMF, >99.0%) was obtained from Samchun Pure Chemical Co. (extra pure grade, Pyeongtaek, Republic of Korea). Anthracene of chemical pure grade, to be used as an internal standard, was obtained from Junsei Chemical Co. (Tokyo, Japan).

Six different phthalates were used for the feasibility study. Dimethyl phthalate (>99.0%), diethyl phthalate (>98.0%), diallyl phthalate (>98.0%), dibutyl phthalate (>97.0%), benzyl butyl phthalate (>97.0%), and di(2-ethylhexyl) phthalate (>98.0%) were obtained from TCI (Tokyo, Japan). The physical properties and chemical structure of all the standard compounds are listed in Table S1. The phthalate stock solutions were prepared with n-hexane (HPLC grade, Samchun Pure Chemical Co., Pyeongtaek, Republic of Korea) at a concentration of 10,000 mg L^−1^, and they were subjected to step-by-step dilution with n-hexane for further use. These solutions were stored in a refrigerator (5 °C) until use. The working solution was used for the experiment by making 20 mg L^−1^ phthalate mixture by diluting each phthalate stock solution. The water used was ultrapure water (18.1 MΩ cm^−1^, Pure Water Co., Namyangju, Republic of Korea).

The homemade INME device consisted of a 22-gauge stainless-steel needle (Hamilton 90,022, metal hub syringe needle, 718 μm O.D., 413 μm I.D., 51 mm length, bevel tip, Hamilton, Reno, NV, USA), a 1 mL Luer lock gas-tight syringe barrel (Hamilton 1001N), and a polytetrafluoroethylene (PTFE Teflon) plunger. The device was used as a headspace device in the needle microextraction (HS–INME) for the adsorption and desorption tests. Stainless-steel wire (0.22 mm O.D.) was obtained from a local company (09one Science, Gyeonggi-do, Republic of Korea) and used as a working electrode for the coulometric coating. The working solution for the coulometric coating was filtrated using a 0.45 μm syringe filter (polyvinylidene fluoride membrane, 25 mm I.D. × 0.45 μm pore size, SV25P045NL, Hyundai Micro, Seoul, Republic of Korea).

### 3.2. Preparation of the MWCNT-IL/PANI Layer Coated on a Wire

MWCNT of 300 mg was refluxed in 21 mL of concentrated nitric acid at 115 °C for 3 h according to a previous report [45]. The resulting oxidized MWCNT solid was filtered through filter paper (No 20, 5 μm~8 μm pore size, Hyundai Micro, Seoul, Republic of Korea), and washed with distilled water until the acidic pH was neutralized; it was then dried at 80 °C overnight. The MWCNT–IL composite was prepared by grinding the mixture of 1.6 mL [HOEMIm] BF4 and 160 mg oxidized MWCNTs with agate mortar [29,46], followed by sonication for 30 min with 18 mL DMF. Then, it was sonicated for an additional 1 h after adding 32 mL distilled water.

The MWCNT–IL/PANI composites were deposited on the surface of a stainless-steel wire using electrochemical polymerization in aqueous solutions containing aniline as a monomer and MWCNT–IL as the carbon nanomaterials, based on a potential difference during the electrochemical polymerization [47]. The combined MWCNT–IL (80 mg) composite was dispersed in 40 mL distilled water for 1 h at room temperature with a sonicator, followed by the addition of 1 mL aniline and further sonication for 15 min. The partially ionizable carboxylic acid groups generated during the oxidation of the MWCNTs with nitric acid can both serve as charge carriers in the solution and charge balance dopants in the polymers. Therefore, no additional supporting electrolyte was used, to avoid penetration of dopants other than the ionized MWCNTs [48].

Coulometric polymerization was performed using a three-electrode arrangement (WonAtech, ZIVE-SP2 electrochemical workstation, Seoul, Republic of Korea) with the filtered MWCNT–IL hybrid composite and an aniline solution at room temperature. Before the electrochemical polymerization step, the stainless-steel wire used as the working electrode was cleaned with acetone, and one end of this wire was coiled up, as mentioned in a previous study [13]. Then, the stainless-steel wire was immersed in the solution; a platinum wire was used as the counter electrode and we proceeded using a RE–5B Ag/AgCl reference electrode (Bioanalytical Systems Inc., W. Lafayette, IN, USA).

**Figure 4:**
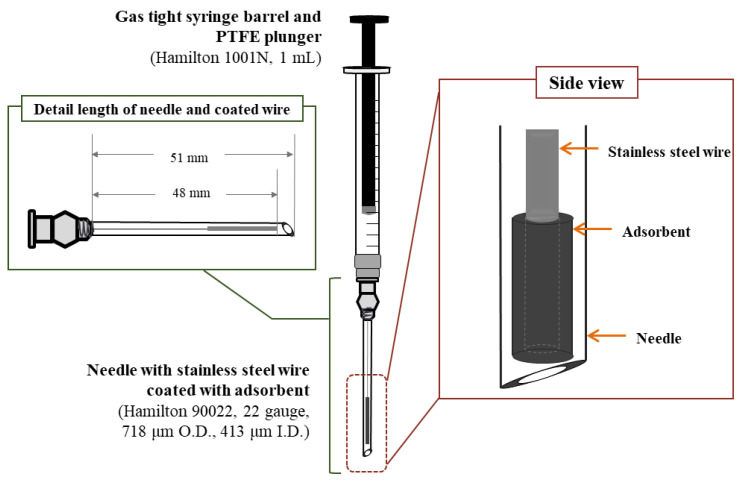
A schematic illustration of the side view and details of each length of a needle containing a stainless-steel wire coated with MWCNT–IL/PANI.

A constant potential at 2.0 V was applied to the electricity measurement system for 500 s. The potential range tested was determined so as to avoid the oxidation of unwanted materials. After the coulometric process, the coating layer was rinsed several times with distilled water to remove unreacted chemicals such as residual MWCNT–IL and aniline. The coated wire was dried in an oven at 80 °C for 30 min and then thermally purified at 230 °C for 1 h. As shown in Figure 4, a wire coated with the MWCNT–IL/PANI adsorbent was inserted into the INME needle. Prior to the extraction test, a needle containing the coated wire was inserted into the GC injector at 230 °C for 30 min for thermal purification, then kept in a desiccator at room temperature. For the extraction process, the INME needle was simply connected to the Luer lock gas-tight syringe barrel and plunger.

### 3.3. Characterization of the MWCNT–IL/PANI Coating Layer

Using the Perkin Elmer Spectrometer 100 (Waltham, MA, USA), FT–IR (Fourier transform infrared) spectra of pristine MWCNT, oxidized MWCNT, and MWCNT–IL complexes were obtained using the KBr pellet method ranging from 450 cm^−1^ to 4000 cm^−1^, and each functional group was identified.

The thermal stability of the MWCNT–IL/PANI layer was determined by thermogravimetric analysis (TGA, SDT Q600, TA Instruments, New Castle, DE, USA). The temperature was raised from room temperature to 800 °C at a heating rate of 10 °C min^−1^ in a nitrogen atmosphere.

The roughness of the adsorbent layer was analyzed using an atomic force microscope (AFM; Park NX10, Suwon, Republic of Korea). The scan area was 20 μm × 20 μm. Then, 3D images of the adsorbent’s upper surface were taken, and the related parameters of surface roughness were obtained using the supporting software. Morphology and energy dispersive spectroscopy (EDS) mapping images of the MWCNT–IL/PANI coating layer surface were obtained using scanning electron microscopy (SEM, Hitachi, SU8230, Tokyo, Japan).

### 3.4. Headspace In–Needle Microextraction Procedure

The test solution prepared for the headspace (HS) extraction test was a mixture of 9.00 mL of water and 1.00 mL of the phthalate working solution in a 50 mL vial. The experiment was performed by exposing the INME needle to the HS of the vial containing the standard solutions. The extraction and adsorption processes were performed by sucking out the analytes in the upper part of the sample vial by automatic compression and aspiration using a homemade high-efficiency extraction reciprocating pump. The speed of the reciprocating pump was 6 cycles/min (10 s/cycle) [49]. After the adsorption was completed, the needle with the analyte adsorbed was immediately connected to another gas-tight syringe and inserted into the GC injection port at 230 °C for 3 min. Then, the analytes were injected into the GC column to be separated at the same time. The INME needle used in the adsorption process was washed with acetone, and the remaining impurities were desorbed after 30 min of conditioning at 230 °C before the next adsorption experiment.

### 3.5. Optimization of HS–INME–MWCNT–IL/PANI

To evaluate the extraction capacity of the MWCNT–IL/PANI coating on a wire for the HS–INME method and to validate the proposed INME method, phthalate standards were saturated in the vial for HS–INME extraction. Various parameters affecting the extraction efficiency were investigated, including the weight/volume percentage, polymerization potential, electrochemical deposition time, and the length of the MWCNT adsorbent for the synthesis of the adsorbent, and the saturation time, extraction time, adsorption time, and desorption time for the HS–INME method. The peak areas of the standard compounds at various conditions were obtained three times and compared for the selection of the optimal conditions based on the amount of adsorption as well as reproducibility. The parameters and conditions for the adsorbent consisting of the MWCNT–IL/PANI layer and the HS–INME method are shown in Table 2.

### 3.6. Validation of HS–INME–MWCNT–IL/PANI

To verify the developed analytical method, a calibration curve measured in triplicate was established to determine the LOD, LOQ, precision, accuracy, and recovery using the phthalate standard solution. Recovery and reproducibility tests were conducted to confirm the accuracy and precision of the analytical method. Quantitative analysis was performed with the internal standard method using anthracene as an internal standard.

The proposed HS–INME–MWCNT–IL/PANI method with GC/MS was used for the feasibility study in the aqueous samples. The aqueous samples were prepared for two commercially available wraps, considering the risk of phthalate influx into food [50]. The wraps investigated in this study were an industrial linear low-density polyethylene (LLDPE) wrap (Okong stretch film, Okong, Republic of Korea) and a household LLDPE wrap (Bio wrap, Comex latex, Republic of Korea). Each wrap (1%, *w*/*v*) was put in water (80 °C, 200 mL) for 3 h and then removed from the water, and an aliquot of the remaining water (9 mL) was taken for phthalate analysis.

### 3.7. Gas Chromatography/Mass Spectrometry (GC/MS)

Optimization and validation of the analytical methods, a comparison of extraction efficiencies, and a quantitative analysis of the real samples were performed using GC/MS. The analyses were carried out using the GC system (Agilent 7820A) and an MSD 5977E Mass Spectrometer in selected ion monitoring (SIM) mode. The separation of phthalates was performed using an HP-5MS analytical column (30 m × 0.25 mm × 0.25 μm, (5%-Phenyl)-methylpolysiloxane, Agilent Technologies, Santa Clara, CA, USA) in splitless mode. Detailed operating conditions of the GC/MS are summarized in Table S2.

## 4. Conclusions

In this study, we successfully synthesized an MWCNT–IL/PANI adsorbent to be coated on stainless steel using the electrochemical polymerization of aniline after combining MWCNTs with IL for INME needle fabrication. The MWCNT–IL hybrid composites showed that the MWCNTs and IL were well-formed from carboxyl groups and an imidazolium cation. As a result, the MWCNT–IL/PANI composite used to coat the stainless-steel wire was evenly coated on the wire’s surface; its high thermal stability was confirmed by the TGA, AFM, and SEM results.

The optimization of the adsorbent design process was performed, and the HS–INME analysis conditions were determined. The optimal weight per volume percent of MWCNTs and IL was 10%, and the MWCNT–IL/PANI composite of 1.0 cm length was effectively electrochemically synthesized on the surface of the stainless-steel wire when a constant potential of 2.0 V was applied for 500 s. The optimum conditions for HS–INME analysis were 60 min of saturation time, a 50 °C extraction temperature, 30 min of adsorption time, and 3 min of desorption time. To validate the HS–INME–MWCNT–IL/PANI, calibration curves, the LOD, the LOQ, the recovery, the reproducibility, and the enrichment factor were determined for optimum conditions using a GC/MS system.

In conclusion, a newly synthesized and characterized MWCNT–IP/PANI was used as an adsorbent for the HS–INME method. The INME needle with an MWCNT–IL/PANI-coated wire layer was used repeatedly up to 150 times without losing performance. This proposed adsorbent and the INME method could be implemented as an environmentally friendly, solvent-free extraction method.

## Figures and Tables

**Table 1:** Validation data of HS–INME using MWCNT–IL/PANI and GC/MS (*n* = 3).

Compound	Regression Equation	Coefficient of Determination (r^2^)	LOD (μg)	LOQ (μg)	Dynamic Range (μg)
Dimethyl phthalate	y = 2.5326x + 0.0233	0.9907	19.57	65.23	65.23~2.50 × 10^2^
Diethyl phthalate	y = 6.5383x + 0.0390	0.9967	15.84	52.79	52.79~1.00 × 10^3^
Diallyl phthalate	y = 2.1476x + 0.0653	0.9959	16.17	53.91	53.91~5.00 × 10^2^
Dibutyl phthalate	y = 5.1304x + 0.1313	0.9734	18.9	63.01	63.01~5.00 × 10^2^
Benzyl butyl phthalate	y = 2.6991x + 0.0557	0.9931	42.03	140.1	140.1~1.00 × 10^3^
Di(2-ethylhexyl) phthalate	y = 2.6567x + 0.0994	0.9918	50.56	168.5	168.5~1.00 × 10^3^

**Table 2:** The parameters of HS–INME using an MWCNT–IL/PANI-coated stainless-steel wire investigated in this study.

MWCNT–IL/PANI Layer Parameter	Conditions
Percentage of MWCNT-ionic liquid (%, *w*/*v*)	5, 10, 15
Polymerization potential (V)	1, 2, 3
Electrochemical deposition time (s)	150, 350, 500, 700
Adsorbent surface length (cm)	0.5, 1.0, 1.5
**HS–INME parameter**	**Conditions**
Saturation time (min)	15, 30, 45, 60
Extraction temperature (°C)	25, 50, 80
Adsorption time (min)	10, 20, 30, 60
Desorption time (min)	0.5, 1, 3, 5

## Data Availability

Not applicable.

## Supplementary Materials

The following supporting information can be downloaded at: https://www.mdpi.com/article/10.3390/molecules28083517/s1, Figure S1: Influence of percentage of MWCNTs and ionic liquid (% *w*/*v*) on the peak area of target analytes; Figure S2: Influence of applied polymerization potential on the peak area of target analytes; Figure S3: Influence of electrochemical deposition time on the peak area of target analytes; Figure S4: Influence of adsorbent surface length on the peak area of target analytes; Figure S5: Influence of saturation time on the peak area of target analytes; Figure S6: Influence of applied extraction temperature on the peak area of target analytes; Figure S7: Influence of adsorption time on the peak area of target analytes; Figure S8. Influence of applied desorption time on the peak area of target analytes; Figure S9. Thermogravimetric analysis curve of MWCNT–IL/PANI adsorbent; Figure S10: AFM images of MWCNTs IL/PANI deposited on the surface of stainless steel wire; Figure S11: Chromatogram obtained from (a) sample 2, and (b) spiked sample 2 by HS INME MWCNT–IL/PANI. Peak 1, dimethyl phthalate; 2, diethyl phthalate; 3, diallyl phthalate; 4, dibutyl phthalate; 5, benzyl butyl phthalate; 6, di(2 ethylhexyl) phthalate; and IS, anthracene; Table S1. Physical properties and chemical structures for each of the phthalates used in the target compounds in this study; Table S2. The operating conditions of gas chromatograph/mass spectrometer (GC/MS); Table S3. Recovery of HS–INME using MWCNT–IL/PANI coating layer followed GC/MS.

## Sample Availability

Not available.

## References

[1] Aly A.A., Górecki T. (2020). Green Approaches to Sample Preparation Based on Extraction Techniques. Molecules.

[2] Câmara J.S., Perestrelo R., Berenguer C.V., Andrade C.F.P., Gomes T.M., Olayanju B., Kabir A., MR Rocha C., Teixeira J.A., Pereira J.A.M. (2022). Green Extraction Techniques as Advanced Sample Preparation Approaches in Biological, Food, and Environmental Matrices: A Review. Molecules.

[3] Picó Y., Pawliszyn J. (2012). Comprehensive Sampling and Sample Preparation.

[4] Jon C.-S., Meng L.-Y., Li D. (2019). Recent review on carbon nanomaterials functionalized with ionic liquids in sample pretreatment application. TrAC Trends Anal. Chem..

[5] Prokůpková G., Holadová K., Poustka J., Hajšlová J. (2002). Development of a solid-phase microextraction method for the determination of phthalic acid esters in water. Anal. Chim. Acta.

[6] Serôdio P., Nogueira J. (2006). Considerations on ultra-trace analysis of phthalates in drinking water. Water Res..

[7] Jobling S., Reynolds T., White R., Parker M.G., Sumpter J.P. (1995). A variety of environmentally persistent chemicals, including some phthalate plasticizers, are weakly estrogenic. Environ. Health Perspect..

[8] Castillo M., Oubina A., Barceló D. (1998). Evaluation of ELISA kits followed by liquid chromatography-atmospheric pressure chemical ionization-mass spectrometry for the determination of organic pollutants in industrial effluents. Environ. Sci. Technol..

[9] Potter D.W., Pawliszyn J. (1994). Rapid determination of polyaromatic hydrocarbons and polychlorinated biphenyls in water using solid-phase microextraction and GC/MS. Environ. Sci. Technol..

[10] Colon I., Dimandja J.D. (2004). High-throughput analysis of phthalate esters in human serum by direct immersion SPME followed by isotope dilution–fast GC/MS. Anal. Bioanal. Chem..

[11] Lee S.Y., Yoon J.H., Bae S., Lee D.S. (2018). In-needle microextraction coupled with gas chromatography/mass spectrometry for the analysis of phthalates generating from food containers. Food Anal. Methods.

[12] Jeon H.L., Son H.H., Bae S., Lee D.S. (2015). Use of polyacrylic acid and polydimethylsiloxane mixture for in-needle microextraction of volatile aroma compounds in essential oils. Bull. Korean Chem. Soc..

[13] Lee E.J., Lee D.S. (2014). Fabrication of in-needle microextraction device using nichrome wire coated with poly(ethylene glycol) and poly(dimethylsiloxane) for determination of volatile compounds in lavender oils. Bull. Korean Chem. Soc..

[14] Bang Y.J., Hwang Y.R., Lee S.Y., Park S.M., Bae S. (2017). Sol–gel-adsorbent-coated extraction needles to detect volatile compounds in spoiled fish. J. Sep. Sci..

[15] Hwang Y., Lee Y., Ahn S., Bae S. (2020). Electrochemically polyaniline-coated microextraction needle for phthalates in water. Anal. Sci. Technol..

[16] Kim S., Bae S., Lee D.-S. (2022). Characterization of scents from Juniperus chinensis by headspace in-needle microextraction using graphene oxide-polyaniline nanocomposite coated wire followed by gas chromatography-mass spectrometry. Talanta.

[17] Kim S., Bae S. (2022). In vitro and in vivo human body odor analysis method using GO:PANI/ZNRs/ZIF-8 adsorbent followed by GC/MS. Molecules.

[18] Motitswe M.G., Badmus K.O., Khotseng L. (2022). Development of Adsorptive Materials for Selective Removal of Toxic Metals in Wastewater: A Review. Catalysts.

[19] Qu L., Dai L. (2005). Substrate-enhanced electroless deposition of metal nanoparticles on carbon nanotubes. J. Am. Chem. Soc..

[20] Randriamahazaka H., Ghilane J. (2016). Electrografting and controlled surface functionalization of carbon based surfaces for electroanalysis. Electroanalysis.

[21] Dobrowolski R., Mróz A., Dąbrowska M., Olszański P. (2017). Solid sampling high-resolution continuum source graphite furnace atomic absorption spectrometry for gold determination in geological samples after preconcentration onto carbon nanotubes. Spectrochim. Acta Part B At. Spectrosc..

[22] Guan Z., Huang Y., Wang W. (2008). Carboxyl modified multi-walled carbon nanotubes as solid-phase extraction adsorbents combined with high-performance liquid chromatography for analysis of linear alkylbenzene sulfonates. Anal. Chim. Acta.

[23] Du W., Zhao F., Zeng B. (2009). Novel multiwalled carbon nanotubes-polyaniline composite film coated platinum wire for headspace solid-phase microextraction and gas chromatographic determination of phenolic compounds. J. Chromatogr. A.

[24] Stege P.W., Lapierre A.V., Martinez L.D., Messina G.A., Sombra L.L. (2011). A combination of single-drop microextraction and open tubular capillary electrochromatography with carbon nanotubes as stationary phase for the determination of low concentration of illicit drugs in horse urine. Talanta.

[25] Jun L.Y., Mubarak N.M., Yee M.J., Yon L.S., Bing C.H., Khalid M., Abdullah E.C. (2018). An Overview of functionalised carbon nanomaterial for organic pollutant removal. J. Ind. Eng. Chem..

[26] Tunckol M., Durand J., Serp P. (2012). Carbon nanomaterial-ionic liquid hybrids. Carbon.

[27] Bellayer S., Gilman J.W., Eidelman N., Bourbigot S., Flambard X., Fox D.M., De Long H.C., Trulove P.C. (2005). Preparation of homogeneously dispersed multiwalled carbon nanotube/polystyrene nanocomposites via melt extrusion using trialkyl imidazolium compatibilizer. Adv. Funct. Mater..

[28] Shim Y., Kim H.J. (2009). Solvation of carbon nanotubes in a room-temperature ionic liquid. ACS Nano.

[29] Li L., Wu M., Feng Y., Zhao F., Zeng B. (2016). Doping of three-dimensional porous carbon nanotube-graphene-ionic liquid composite into polyaniline for the headspace solid-phase microextraction and gas chromatography determination of alcohols. Anal. Chim. Acta.

[30] Wu M., Zhang H., Zhao F., Zeng B. (2014). A novel poly(3,4-ethylenedioxythiophene)-ionic liquid composite coating for the headspace solid-phase microextraction and gas chromatography determination of several alcohols in soft drinks. Anal. Chim. Acta.

[31] Fukushima T., Kosaka A., Ishimura Y., Yamamoto T., Takigawa T., Ishii N., Aida T. (2003). Molecular ordering of organic molten salts triggered by single-walled carbon nanotubes. Science.

[32] Pawliszyn J. (2000). Theory of solid-phase microextraction. J. Chromatogr. Sci..

[33] Ping Z. (1996). In situ FT-IR-attenuated total reflection spectroscopic investigations on the base-acid transitions of polyaniline. base-acid transition in the emeraldine form of polyaniline. J. Chem. Soc. Faraday Trans..

[34] Furukawa Y., Ueda F., Hyodo Y., Harada I., Nakajima T., Kawagoe T. (1988). Vibrational spectra and structure of polyaniline. Macromolecules.

[35] Trchová M., Stejskal J. (2011). Polyaniline: The infrared spectroscopy of conducting polymer nanotubes (IUPAC technical report). Pure Appl. Chem..

[36] Boyer M.I., Quillard S., Rebourt E., Louarn G., Buisson J.P., Monkman A., Lefrant S. (1998). Vibrational Analysis of Polyaniline: A model compound approach. J. Phys. Chem. B.

[37] Zheng W., Angelopoulos M., Epstein A.J., MacDiarmid A.G. (1997). Experimental evidence for hydrogen bonding in polyaniline: Mechanism of aggregate formation and dependency on oxidation state. Macromolecules.

[38] Tammer M.G. (2004). Sokrates: Infrared and Raman characteristic group frequencies: Tables and charts. Colloid Polym. Sci..

[39] Dhand C., Arya S.K., Singh S.P., Singh B.P., Datta M., Malhotra B. (2008). Preparation of polyaniline/multiwalled carbon nanotube composite by novel electrophoretic route. Carbon.

[40] Ngo C.L., Le Q.T., Ngo T.T., Nguyen D.N., Vu M.T. (2013). Surface modification and functionalization of carbon nanotube with some organic compounds. Adv. Nat. Sci. Nanosci. Nanotechnol..

[41] Kumar B.R., Rao T.S. (2012). AFM Studies on Surface Morphology, Topography and Texture of Nanostructured Zinc Aluminum Oxide Thin Films. Dig. J. Nanomater. Biostruct..

[42] Thompson M., Ellison S.L.R., Wood R. (2002). Harmonized guidelines for single-laboratory (IUPAC technical report). Pure Appl. Chem..

[43] Currie L.A. (1995). Nomenclature in evaluation of analytical methods including detection and quantification capabilities. Pure Appl. Chem..

[44] Fasano E., Bono-Blay F., Cirillo T., Montuori P., Lacorte S. (2012). Migration of phthalates, alkylphenols, bisphenol A and di(2-ethylhexyl)adipate from food packaging. Food Control.

[45] Asadollahzadeh H., Noroozian E., Maghsoudi S. (2010). Solid-phase microextraction of phthalate esters from aqueous media by electrochemically deposited carbon nanotube/polypyrrole composite on a stainless steel fiber. Anal. Chim. Acta.

[46] Sun Y., Fang Z., Wang C., Zhou A., Duan H. (2015). Incorporating nanoporous polyaniline into layer-by-layer ionic liquid-carbon nanotube-graphene paper: Towards freestanding flexible electrodes with improved supercapacitive performance. Nanotechnology.

[47] El Rhazi M., Majid S., Elbasri M., Salih F.E., Oularbi L., Lafdi K. (2018). Recent progress in nanocomposites based on conducting polymer: Application as electrochemical sensors. Int. Nano Lett..

[48] Chen G.Z., Shaffer M.S.P., Coleby D., Dixon G., Zhou W., Fray D.J., Windle A.H. (2000). Carbon nanotube and polypyrrole composites: Coating and doping. Adv. Mater..

[49] Son H.H., Bae S., Lee D.S. (2012). New needle packed with polydimethylsiloxane having a micro-bore tunnel for headspace in-needle microextraction of aroma components of citrus oils. Anal. Chim. Acta.

[50] Qian S., Ji H., Wu X.X., Li N., Yang Y., Bu J., Zhang X., Qiao L., Yu H., Xu N. (2018). Detection and quantification analysis of chemical migrants in plastic food contact products. PLoS ONE.

