# Supplementary material for: Synthesis and Characterization of a Multi-Walled Carbon Nanotube–Ionic Liquid/Polyaniline Adsorbent for a Solvent-Free In-Needle Microextraction Method
Source: Molecules. 2023 Apr 17;28(8):3517. doi: 10.3390/molecules28083517 (PMC10142705; doi:10.3390/molecules28083517)
Supplement: Supplementary file 1 [file molecules-28-03517-s001.zip › molecules-2193073-supplementary.pdf]

Supplementary materials for

Synthesis and Characterization of a Multi-Walled Carbon Nanotube-Ionic Liquid/ Polyaniline Adsorbent for a Solvent-Free In Needle Microextraction Method

Soyoung Ahn, Sunyoung Bae*

Department of Chemistry, Seoul Women’s University, 621 Hwarang-ro, Nowon-gu, Seoul 01797, Republic of Korea

*Corresponding Author

Email: sbae@swu.ac.kr

Table S1. Physical properties and chemical structures for each of the phthalates used in the target compounds in this study

Compound (Abbreviation)	Chemical formula	Molecular weight (g/mol)	Density (g/mL)	Purity (%)	Chemical structure
Dimethyl phthalate (DMP)	$C_{10}H_{10}O_4$	194.19	1.19	99.0	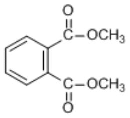
Diethyl phthalate (DEP)	$C_{12}H_{14}O_4$	222.24	1.12	98.0	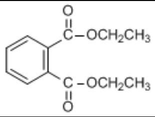
Diallyl phthalate (DAP)	$C_{14}H_{14}O_4$	246.26	1.12	98.0	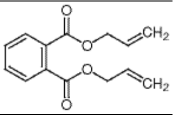
Dibutyl phthalate (DBP)	$C_{16}H_{22}O_4$	278.35	1.05	97.0	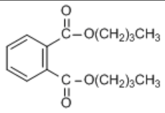
Benzyl butyl phthalate (BBP)	$C_{19}H_{20}O_4$	312.37	1.12	97.0	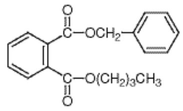
Di(2-ethylhexyl) phthalate (DEHP)	$C_{24}H_{38}O_4$	390.56	0.99	98.0	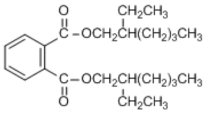

Table S2. The operating conditions of gas chromatograph/mass spectrometer (GC/MS)

	**GC (7820A, Agilent) MS (5977E, Agilent)**
**Column**	HP-5® (30 m x 0.25 mm x 0.25 µm, (5%-Phenyl)-methylpolysiloxane, Agilent)
**Oven temperature program**	60°C → 20°C/min → 280°C (5 min)
**Injector temperature**	230°C
**Injector mode**	splitless
**Carrier gas**	He (99.999%), 1 mL/min
**Mass Detector**	Mass transfer line: 280°C, Ionization voltage: 70 eV<br>Ion source: 230°C, Quadruple: 150°C<br>Scan mode/ Selected Ion Monitoring (SIM) mode

Table S3. Recovery of HS−INME using MWCNTs−IL/PANI coating layer followed GC/MS

Analytes	Sample 1 Industrial use		Sample 2 Food application		Reproducibility (± RSD%, n=5)	
	Detected concentration (µg)	Recovery[a] (%)	Detected concentration (µg)	Recovery[a] (%)	Intra assay (run to run)	Inter assay (needle to needle, 5 needles)
Dimethyl phthalate	< LOD	107.59 ± 4.83[c]	< LOD	95.72 ± 4.51	10.23	17.45
Diethyl phthalate	< LOD	106.11± 8.36	< LOD	96.17± 5.20	10.06	11.49
Diallyl phthalate	< LOD	108.21 ± 6.81	< LOD	92.91 ± 14.90	5.30	13.74
Dibutyl phthalate	26.05 ± 3.12[b]	61.13 ± 4.92	24.87± 1.65	56.58 ± 5.49	7.70	15.81
Benzyl butyl phthalate	< LOD	69.77 ± 15.13	< LOD	56.57± 8.46	12.11	17.22
Di(2-ethylhexyl) phthalate	< LOD	68.91 ± 17.85	< LOD	57.48 ± 17.13	11.92	20.00

[a] Recovery data for spiked with 2.00×10^{2} µg phthalate

[b] Mean value ± standard deviation (n = 3)

[c] Recovery ± RSD (%) (n = 3)

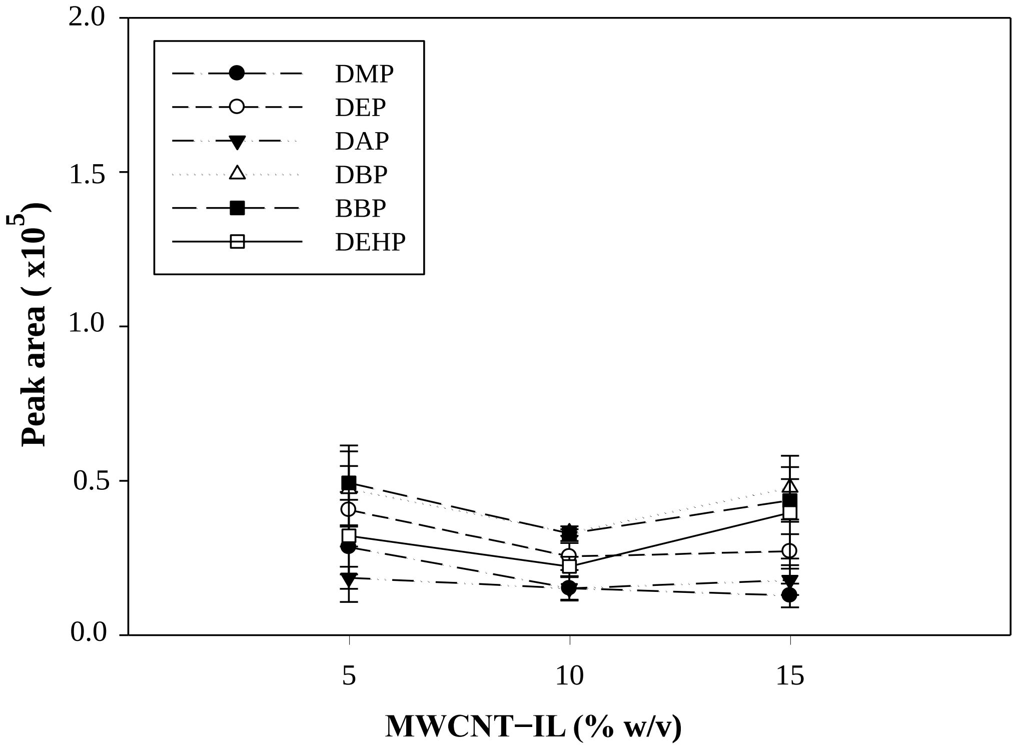

Figure S1. Influence of percentage of MWCNTs and ionic liquid (% w/v) on the peak area of target analytes. The standard deviation (n=3) is represented by a bar. HS−INME−MWCNT−IL/PANI conditions: polymerization potential 2.0 V, polymerization time 500 s, coating layer length of 1 cm, saturation time 60 min, extraction at 50°C, adsorption time for 30 min, and desorption at 230°C for 3 min.

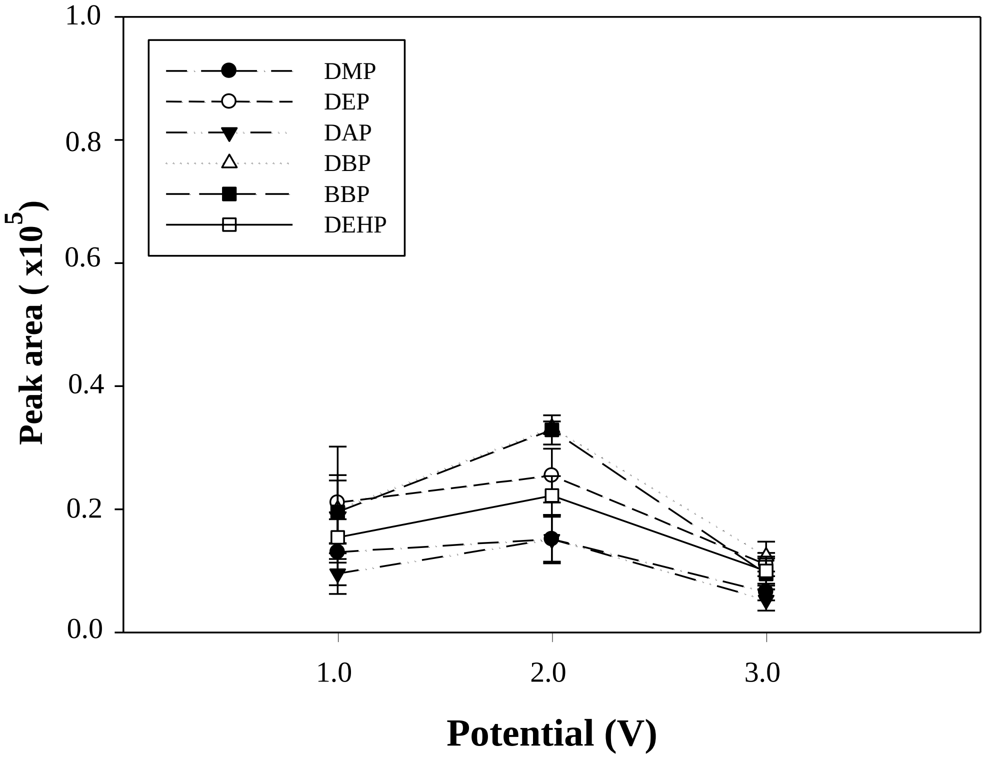

Figure S2. Influence of applied polymerization potential on the peak area of target analytes. The standard deviation (n=3) is represented by a bar. HS−INME−MWCNT−IL/PANI conditions: 10% MWCNTs−IL (% w/v), polymerization time 500 s, coating layer length of 1 cm, saturation time 60 min, extraction at 50°C, adsorption time for 30 min, and desorption at 230°C for 3 min.

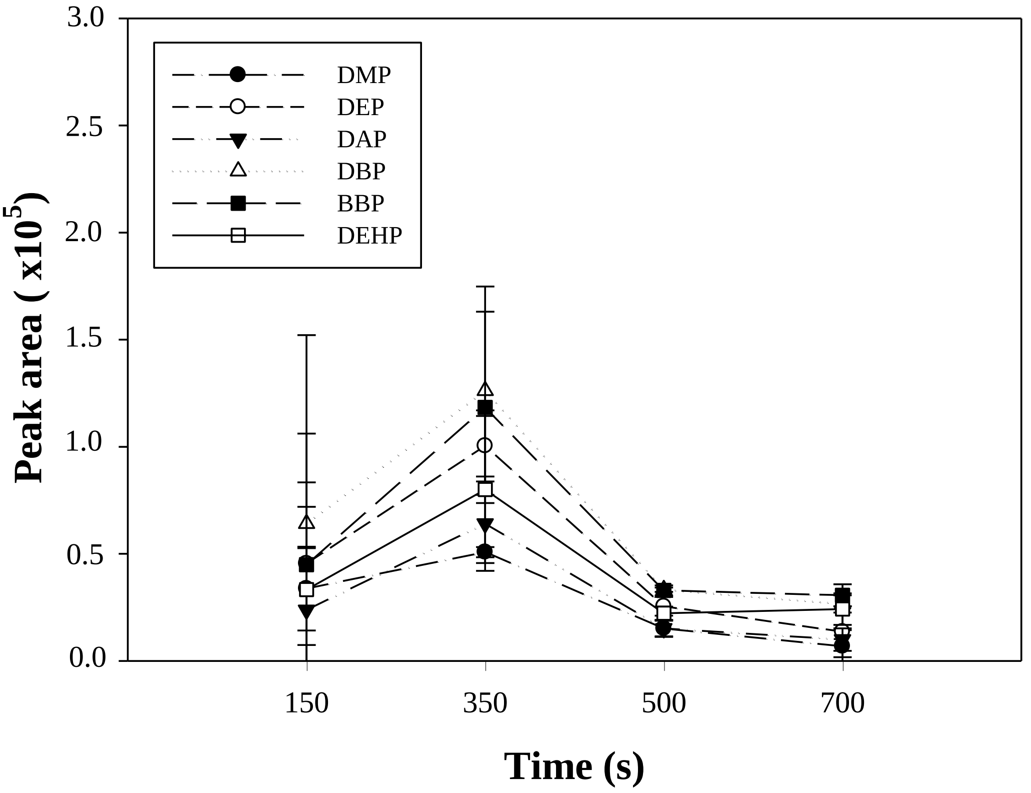

Figure S3. Influence of electrochemical deposition time on the peak area of target analytes. The standard deviation (n=3) is represented by a bar. HS−INME−MWCNT−IL/PANI conditions: 10% MWCNT−IL (% w/v), polymerization potential 2.0 V, coating length of 1 cm, saturation time 60 min, extraction at 50°C, adsorption time for 30 min, and desorption at 230°C for 3 min.

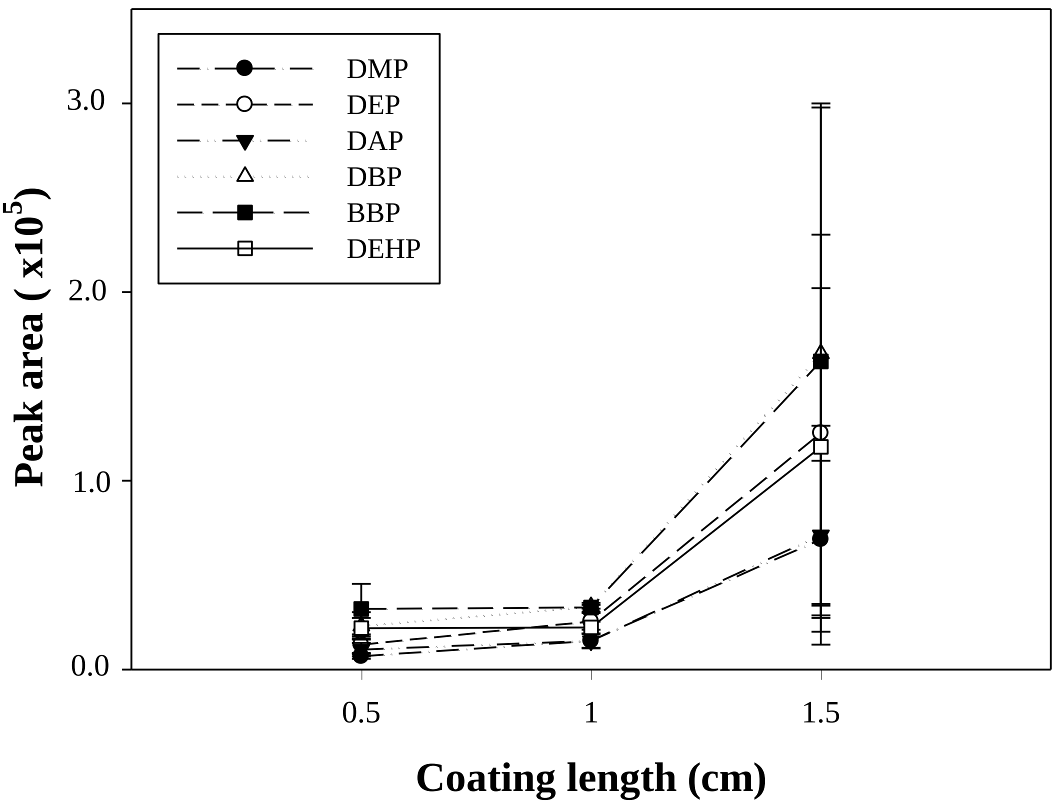

Figure S4. Influence of adsorbent surface length on the peak area of target analytes. The standard deviation (n=3) is represented by a bar. HS−INME−MWCNT−IL/PANI conditions: 10% MWCNT−IL (% w/v), polymerization potential 2.0 V, polymerization time 500 s, saturation time 60 min, extraction at 50°C, adsorption time for 30 min, and desorption at 230°C for 3 min.

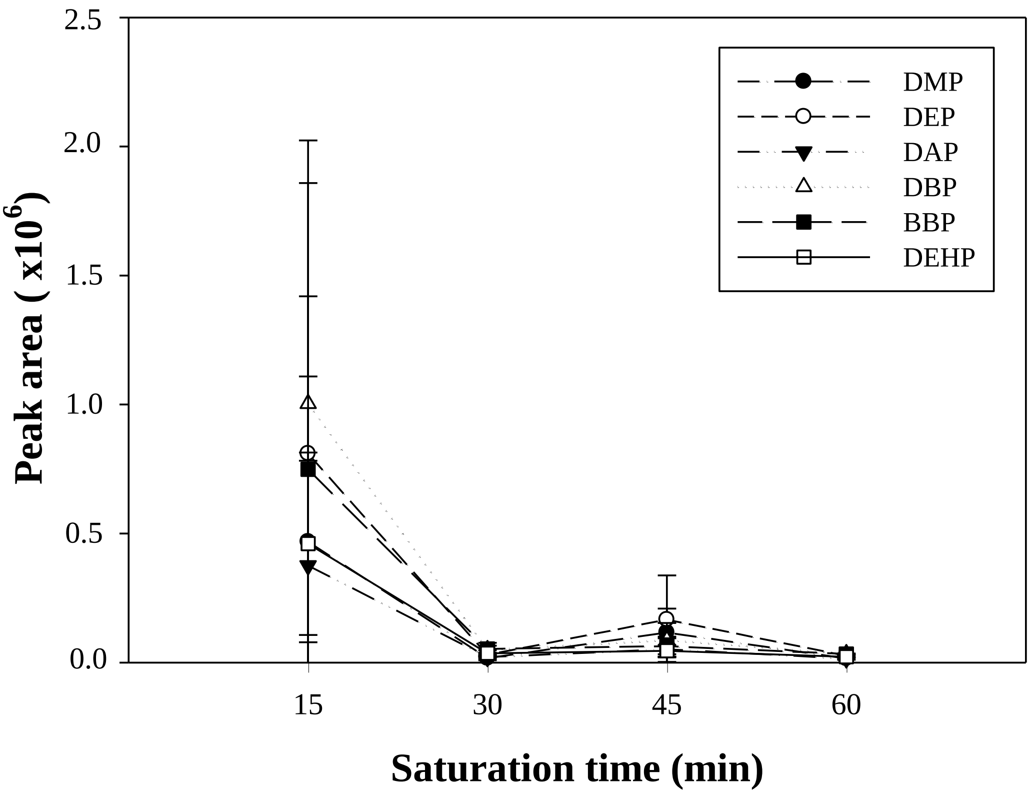

Figure S5. Influence of saturation time on the peak area of target analytes. The standard deviation (n=3) is represented by a bar. HS−INME−MWCNT−IL/PANI conditions: 10% MWCNT−IL (% w/v), polymerization potential 2.0 V, polymerization time 500 s, coating layer length of 1 cm, extraction at 50°C, adsorption time for 30 min, and desorption at 230°C for 3 min.

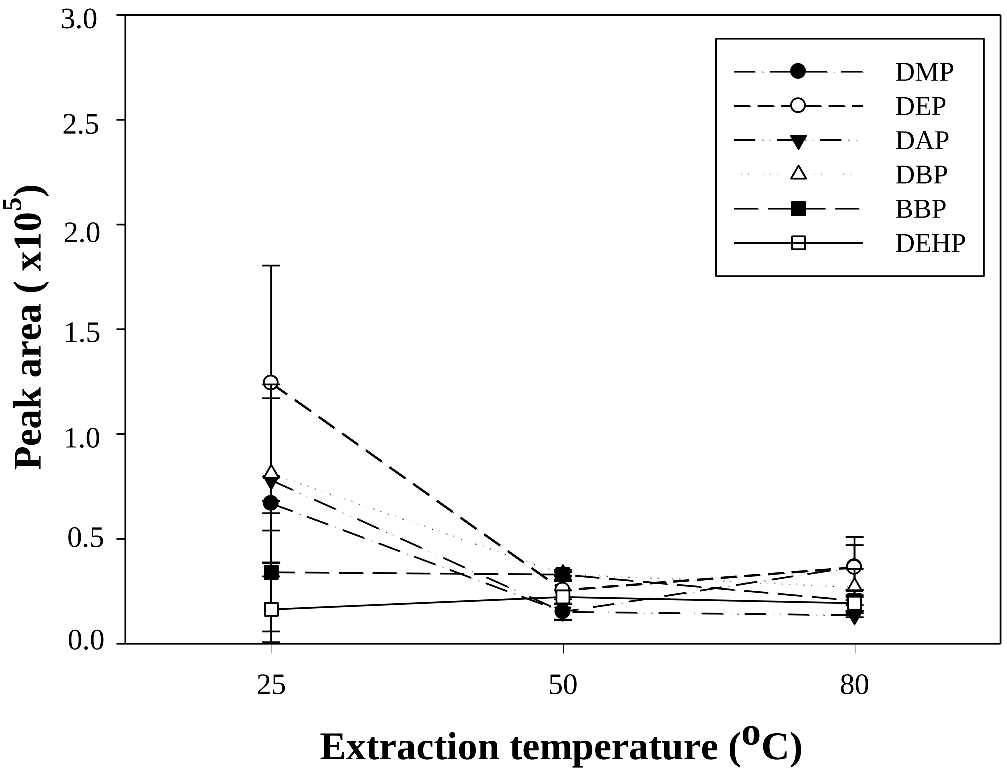

Figure S6. Influence of applied extraction temperature on the peak area of target analytes. The standard deviation (n=3) is represented by a bar. HS−INME−MWCNT−IL/PANI conditions: 10% MWCNT−IL (% w/v), polymerization potential 2.0 V, polymerization time 500 s, coating layer length of 1 cm, saturation time 60 min, adsorption time for 30 min, and desorption at 230°C for 3 min.

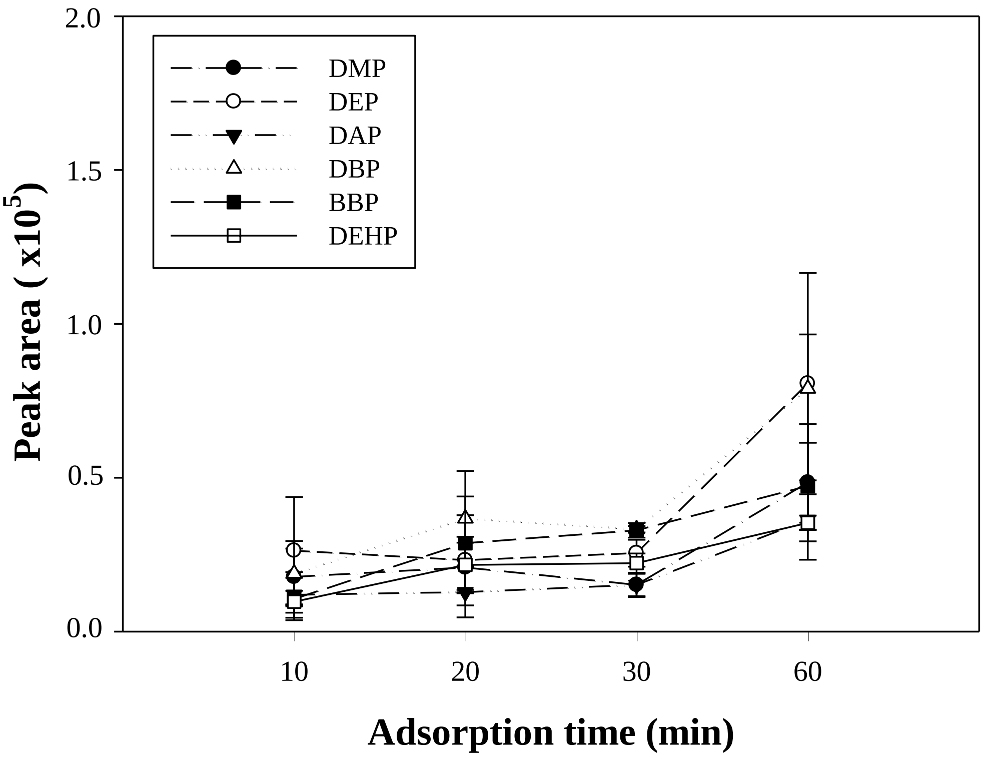

Figure S7. Influence of adsorption time on the peak area of target analytes. The standard deviation (n=3) is represented by a bar. HS−INME−MWCNT−IL/PANI conditions: 10% MWCNT−IL (% w/v), polymerization potential 2.0 V, polymerization time 500 s, coating layer length of 1 cm, saturation time 60 min, extraction at 50°C, and desorption at 230°C for 3 min.

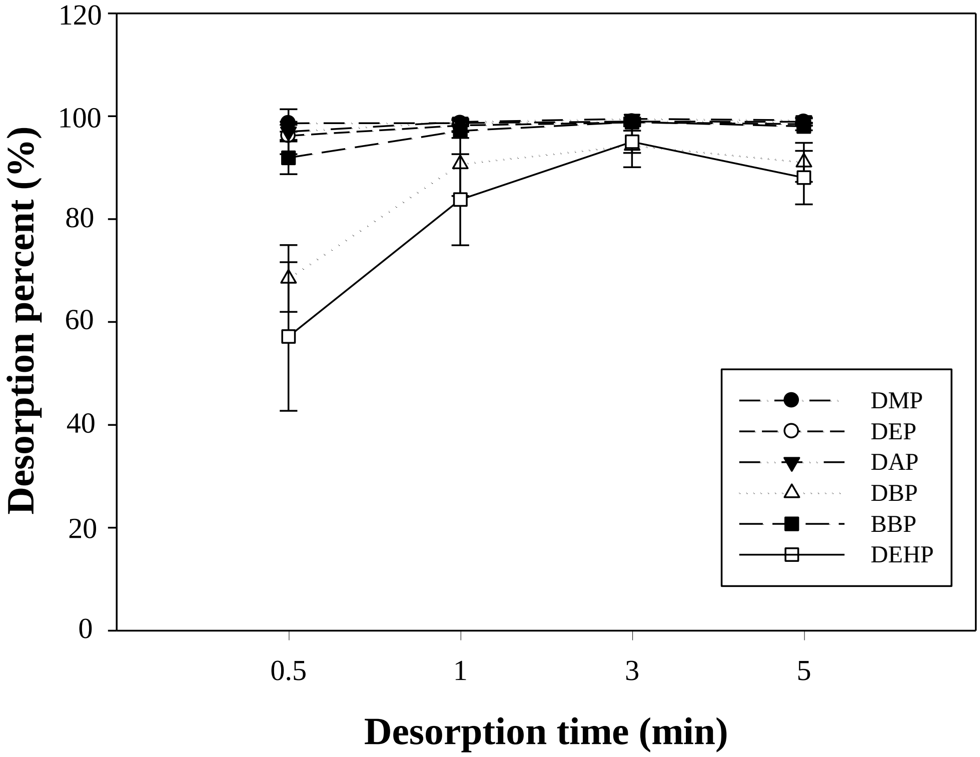

Figure S8. Influence of applied desorption time on the peak area of target analytes. The standard deviation (n=3) is represented by a bar. HS−INME−MWCNT−IL/PANI conditions: 10% MWCNT−IL (% w/v), polymerization potential 2.0 V, polymerization time 500 s, coating layer length of 1 cm, saturation time 60 min, extraction at 50°C, adsorption time for 30 min, and desorption at 230°C.

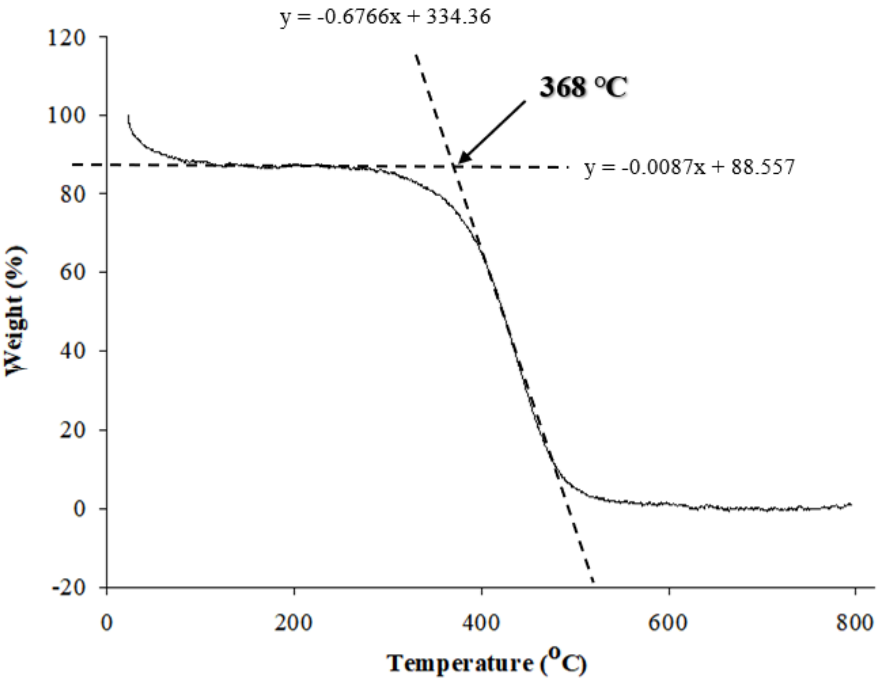

Figure S9. Thermogravimetric analysis curve of MWCNT−IL/PANI adsorbent.

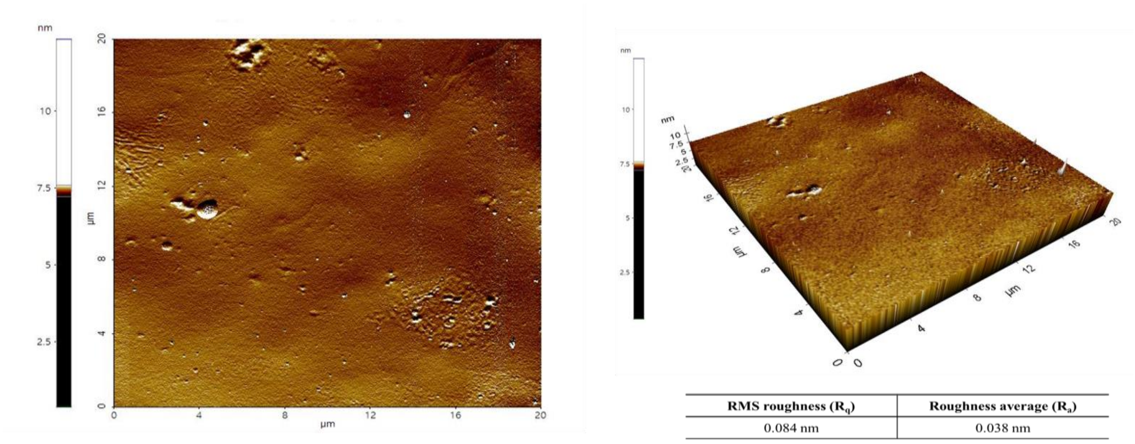

RMS roughness (R_q)	Roughness average (R_a)
0.084 nm	0.038 nm

Figure S10. AFM images of MWCNT−IL/PANI deposited on the surface of stainless steel wire.

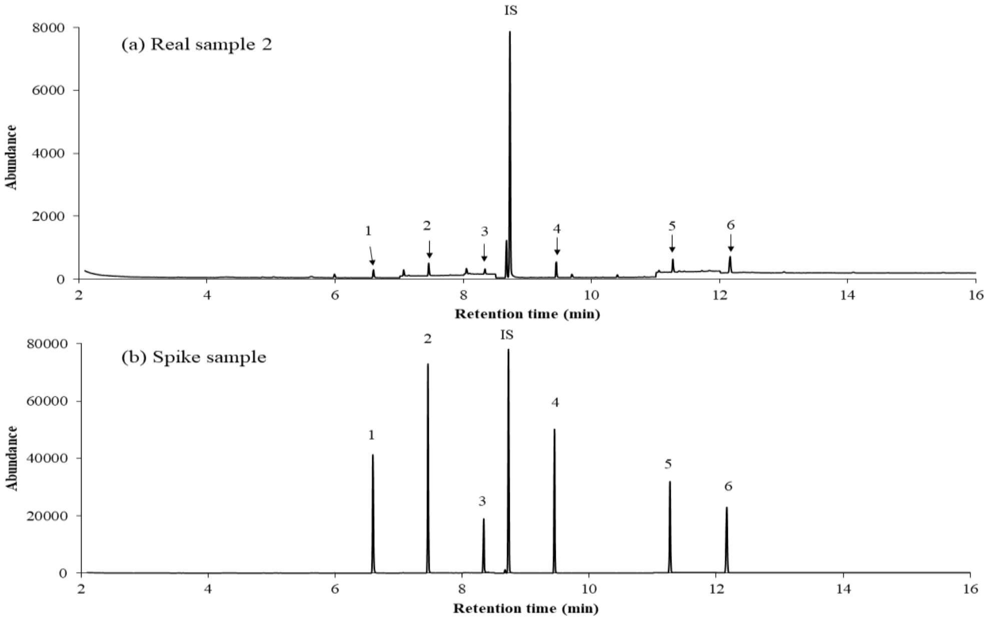

Figure S11. Chromatogram obtained from (a) sample 2, and (b) spiked sample 2 by HS–INME–MWCNT–IL/PANI. Peak 1, dimethyl phthalate; 2, diethyl phthalate; 3, diallyl phthalate; 4, dibutyl phthalate; 5, benzyl butyl phthalate; 6, di(2-ethylhexyl) phthalate; and IS, anthracene

.
